# Supplementary material for: Visible light-exposed lignin facilitates cellulose solubilization by lytic polysaccharide monooxygenases
Source: Nat Commun. 2023 Feb 24;14:1063. doi: 10.1038/s41467-023-36660-4 (PMC9958194; doi:10.1038/s41467-023-36660-4)
Supplement: Supplementary file 1 — Supplementary Information [file 41467_2023_36660_MOESM1_ESM.pdf]

Supplementary information

Visible light-exposed lignin facilitates cellulose solubilization by lytic polysaccharide monooxygenases

Eirik G. Kommedal[1], Camilla F. Angeltveit[1], Leesa J. Klau[2], Iván Ayuso-Fernández[1], Bjørnar Arstad[3], Simen G. Antonsen[1], Yngve Stenstrøm[1], Dag Ekeberg[1], Francisco Gírio[4], Florbela Carvalheiro[4], Svein J. Horn[1], Finn Lillelund Aachmann[2], Vincent G. H. Eijsink[1*]

[1] Faculty of Chemistry, Biotechnology and Food Science, Norwegian University of Life Sciences (NMBU), 1432 Ås, Norway

[2] Department of Biotechnology and Food Science, Norwegian University of Science and Technology (NTNU), 7491 Trondheim, Norway

[3] SINTEF Industry, Process Chemistry and Functional Materials, 0373 Oslo, Norway

[4] National Laboratory of Energy and Geology (LNEG), 1649-038 Lisboa, Portugal

[*] Corresponding author: vincent.eijsink@nmbu.no

The supplementary information includes

Supplementary Figures

Supplementary Figure 1. Probing for LPMO inactivation.

Supplementary Figure 2. Comparison of H_2O_2 consumption in standard dark reaction conditions in the presence or absence of LPMO.

Supplementary Figure 3. The effect of Avicel on light-driven H_2O_2 production in the absence of LPMO.

Supplementary Figure 4. Lignin-driven AA9 activity on cellulose.

Supplementary Figure 5. Kinetic traces of lignin oxidation by bacterial LPMOs.

Supplementary Figure 6. Lignin-driven *Sm*AA10A activity on β-chitin.

Supplementary Figure 7. Light-induced changes in kraft lignin assessed by 1D proton NMR spectroscopy.

Supplementary Figure 8. Light-induced and LPMO-induced changes in organosolv birch lignin assessed by 1D carbon NMR spectroscopy.

Supplementary Figure 9. Light-induced and LPMO-induced changes in organosolv spruce lignin assessed by 2D HSQC NMR spectroscopy.

Supplementary Figure 10. **Light-induced and LPMO-induced changes in organosolv birch lignin assessed by 2D HSQC NMR spectroscopy.**

Supplementary Figure 11. Light-induced and LPMO-induced changes in organosolv birch and spruce lignin assessed by 1D proton NMR spectroscopy.

Supplementary Figure 12. UV-Vis absorption spectra of kraft lignin before and after dialysis.

Supplementary figure 13. Chromatographic analysis of oxidized products generated in anaerobic LPMO reactions with visible light-exposed lignin.

Supplementary Figure 14. Verification of Superoxide Dismutase (SOD) activity.

Supplementary references

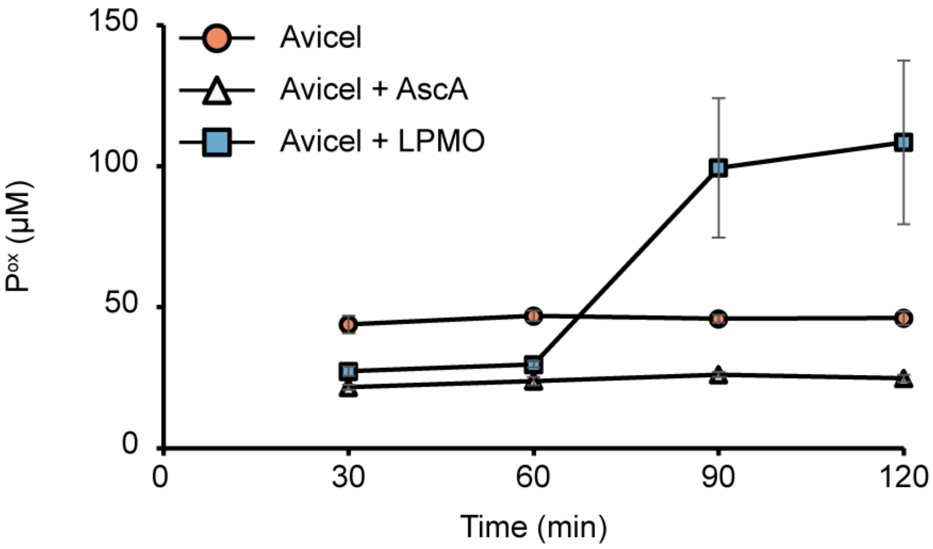

Supplementary Figure 1. Probing for LPMO inactivation. The graphs show time-courses for the release of aldonic acid products. All reactions were carried out with similar initial conditions: Avicel (10 $g.L^{-1}$), Kraft lignin (9 $g.L^{-1}$) and *Sc*AA10C (75 nM) in sodium phosphate buffer (50 mM, pH 7.0) at 40°C under magnetic stirring and exposed to visible light (I=10% I_{max}, approx. 16.8 $W.cm^{-2}$). After 60 min, Avicel (2.3 $g.L^{-1}$), Avicel (2.3 $g.L^{-1}$) and LPMO (100 nM), or Avicel (2.3 $g.L^{-1}$) and reductant (2.3 mM) were added to separate reactions, as indicated in the Figure. Upon sampling, reactions were stopped by filtration, separating the LPMO from its substrate. Before product quantification, solubilized cello-oligosaccharides were hydrolyzed with *Tf*Cel6A to convert LPMO products, with varying degree of polymerization (DP), to a mixture of DP 2 and 3 [GlcGlc1A, (Glc)2Glc1A], the amounts of which were summed up to yield the concentration of solubilized oxidized sites. The data is presented as mean values and error bars show ± s.d. (n = 3, independent experiments).

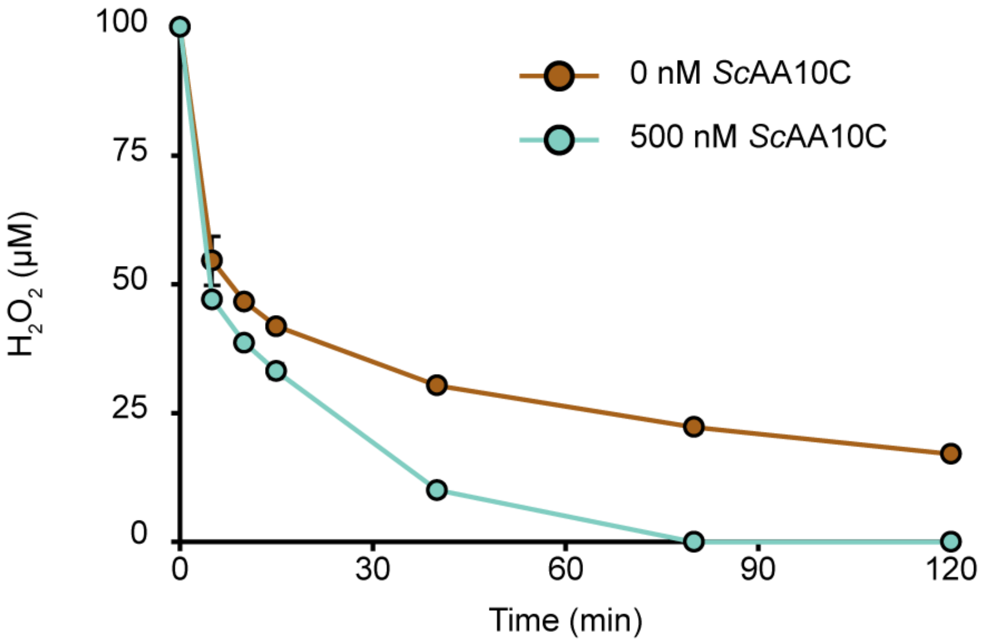

Supplementary Figure 2. Comparison of H_2O_2 consumption in standard dark reaction conditions in the presence or absence of LPMO. The graph shows time courses for consumption of H_2O_2 (added to 100 µM at t = 0) in the presence or absence of *Sc*AA10C (0.5 µM) in reactions with Avicel (10 g.L^{-1}) and kraft lignin (0.9 g.L^{-1}) in sodium phosphate buffer (50 mM, pH 7.0) at 40°C under magnetic stirring in the dark. The curves show that after an initial phase of equally fast H_2O_2 consumption lasting some 30 min, the reaction with the LPMO leads to faster H_2O_2 consumption in the later phase of the reaction (note that the LPMO reactions reported in the manuscript typically lasted 6 hours). The data points represent the mean of three independent experiments and error bars show ± s.d (n = 3).

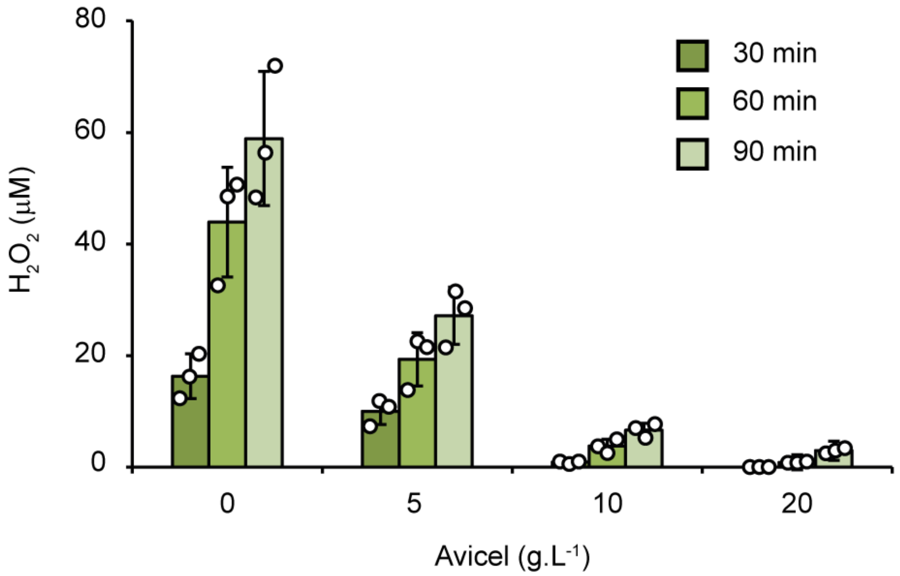

Supplementary Figure 3. The effect of Avicel on light-driven H_2O_2 production in the absence of LPMO. The graphs show time-courses for production of H_2O_2 in photobiocatalytic reactions containing lignin (0.9 g/L) and varying concentrations of Avicel. All reactions were carried out in sodium phosphate buffer (50 mM, pH 7.0) at 40 ºC under magnetic stirring with exposure to visible light (I=10 % I_{max}, approx., 16.8 $W.cm^{-2}$). H_2O_2 accumulation was measured as indicated in the main manuscript methods. The data is presented as mean values and error bars show ± s.d. (n = 3, independent experiments).

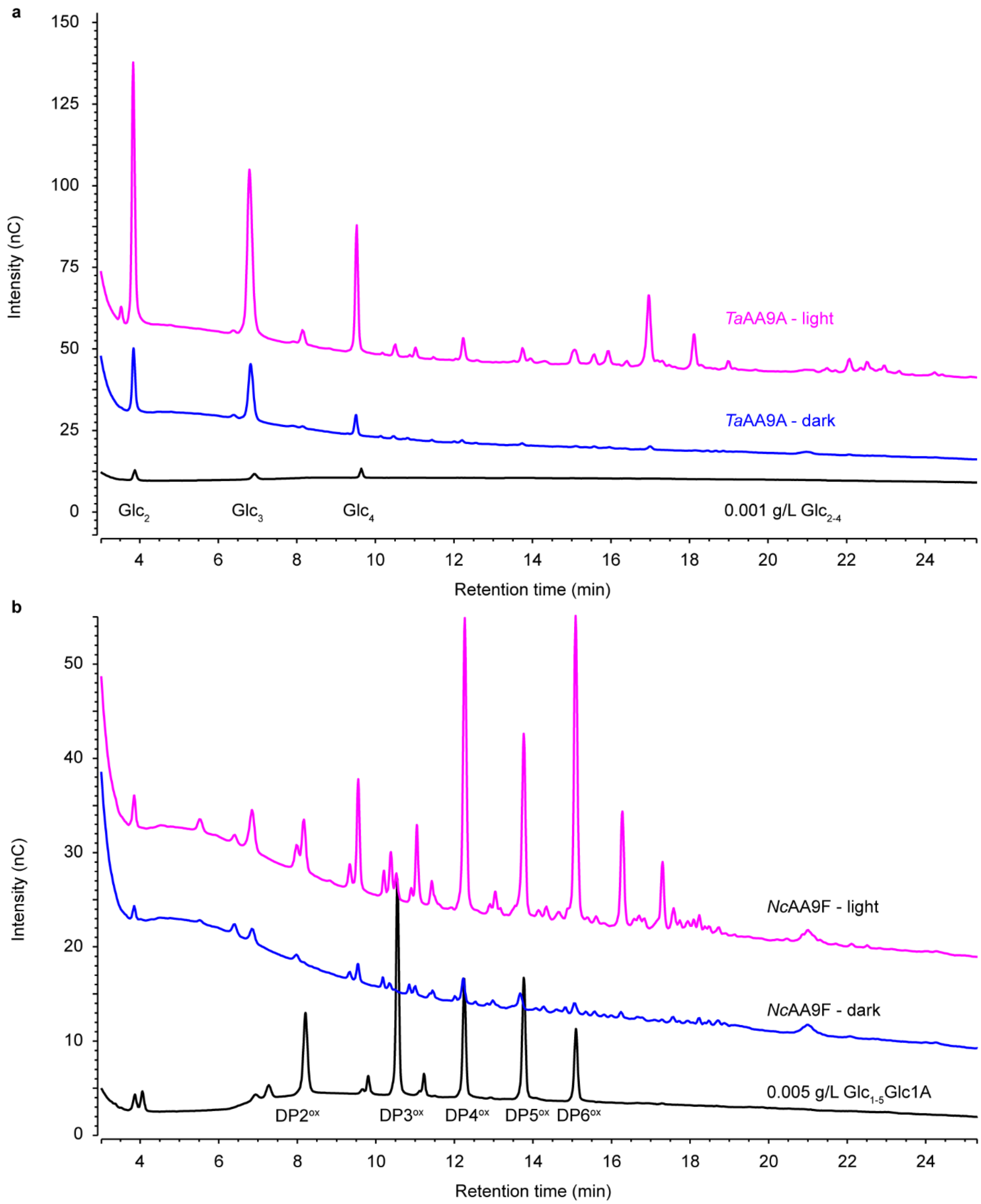

Supplementary Figure 4. Lignin-driven AA9 activity on cellulose. The figure shows chromatographic product profiles obtained for reactions containing kraft lignin (0.9 g.L^{-1}), Avicel (10 g.L^{-1}), and (**a**) *Ta*AA9A (0.5 µM) or (**b**) *Nc*AA9F (0.5 µM) after 6 h of reaction time. All reactions were performed in Bis-Tris buffer (50 mM, pH 7.0) at 40°C under magnetic stirring with or without light-exposure (I=10% I_{max}, approx. 16.8 W.cm^{-2}). All reactions were performed as two independent replicates (n = 2) and a representative product profile is shown. *Ta*AA9A is a primarily C4-oxidizing LPMO and, due to the nature of the analytics[1], its products are converted to native cello-oligomers; hence, the standard in the upper panel (black chromatogram) shows a mixture of native cello-oligomers (Glc_{2-4}). *Nc*AA9F is a C1-oxidizing LPMO; hence the standard in the lower panel (black chromatogram) shows a mixture of

C1-oxidized cello-oligomers ($Glc_{1-5}Glc1A$, referred to as $DP2^{ox}$ - $DP6^{ox}$). Both panels show that irradiation with visible light (upper chromatogram, magenta) increases product formation relative to the corresponding reaction in the dark (middle chromatogram, blue).

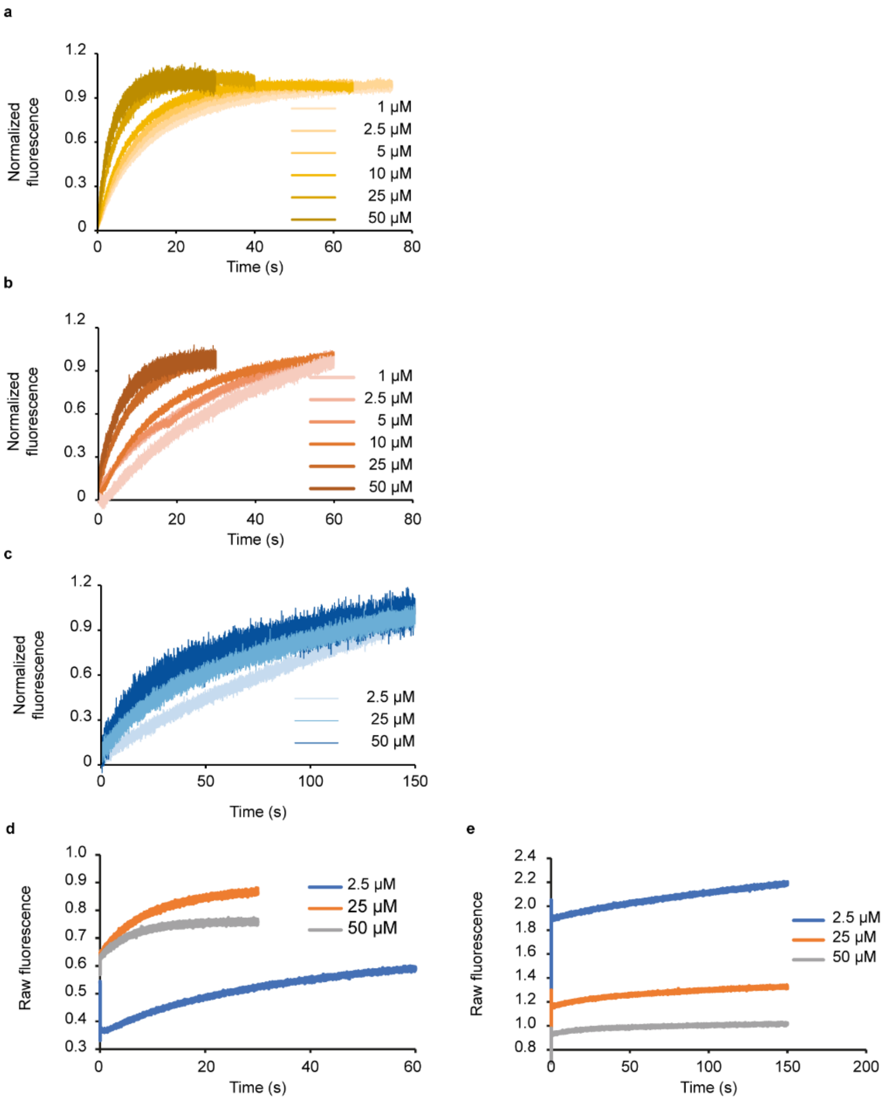

Supplementary Figure 5. Kinetic traces of lignin oxidation by bacterial LPMOs. The figure shows representative fluorescence traces for *Sm*AA10A (CBP21; chitin-active) catalyzed oxidation of native (a) and dialyzed (b) kraft lignin, and *Sc*AA10C (CelS2; cellulose-active) catalyzed oxidation of dialyzed kraft lignin (c). LPMO-Cu(II) (5 μM, final concentration after mixing) was anaerobically mixed with varying concentrations of kraft lignin and the change in fluorescence following reduction of LPMO-Cu(II) to LPMO-Cu(I) was monitored over time. The fluorescence signal was normalized as $F_N = (F_{max}-F(t))/(F_{max}-F_0)$, where F_{max} and F_0 are the fluorescence of the reduced and the oxidized LPMOs, respectively. All reactions were carried out in sodium phosphate buffer (50 mM; pH 7.0) at 25°C. Each experiment was performed in triplicates (n = 3) and a representative replicate is shown. Panels d and e show examples of the underlying raw data for reactions with dialyzed kraft lignin with *Sm*AA10A (corresponding to panel b) and *Sc*AA10C (corresponding to panel c), respectively.

Reliable data could only be obtained for the chitin-active LPMO, *Sm*AA10A, for which the difference in fluorescence signal between ground state and reduced copper state is higher, compared to *Sc*AA10C. Since the reactions contained spectroscopically active lignin, such a strong signal was needed to obtain reliable data. The stronger signal of *Sm*AA10A is likely due to the following: (1) *Sm*AA10A has more tryptophans near the copper ion; this improves signal strength; (2) *Sc*AA10C has an additional domain that contains tryptophans which give a high "background" fluorescence signal that is less affected by the redox state of the copper. Although no reliable rates could be obtained for cellulose-active *Sc*AA10C, comparison of panel c with panels a an b suggests that reduction of this enzyme is slower than reduction of *Sm*AA10A.

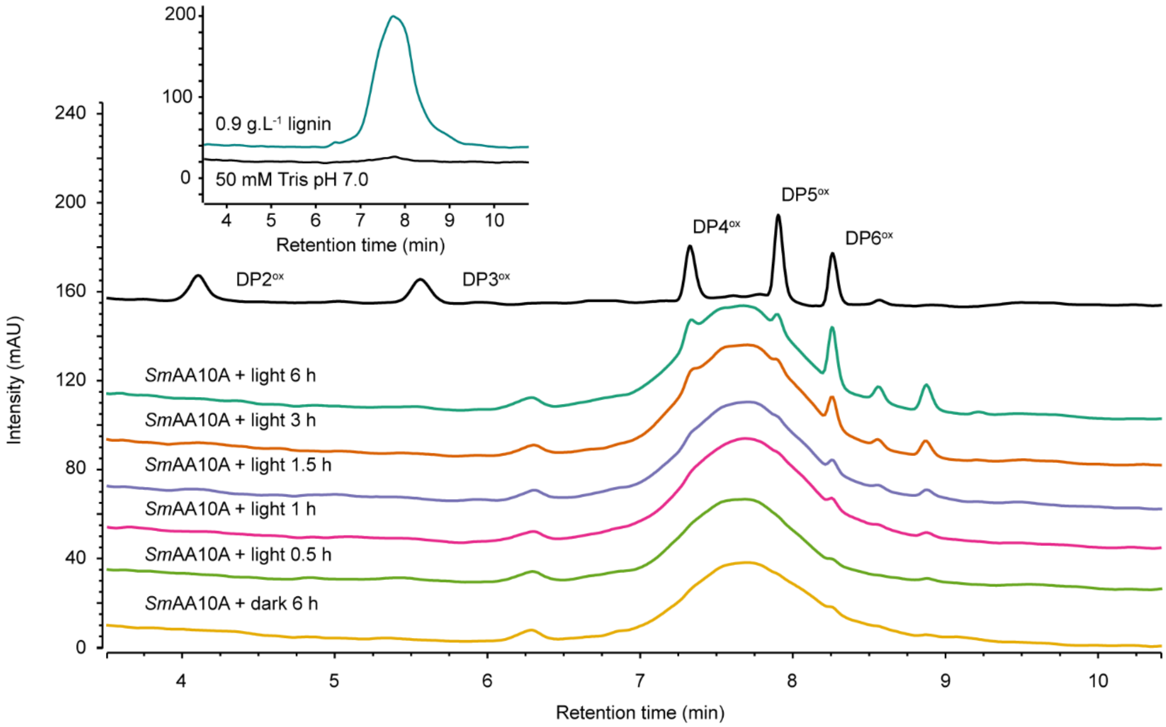

Supplementary Figure 6. Lignin-driven *Sm*AA10A activity on β-chitin. The figure shows chromatographic product profiles obtained for reactions with or without light-exposure containing kraft lignin (0.9 $g.L^{-1}$), β-chitin (10 $g.L^{-1}$), and *Sm*AA10A (0.5 µM). All reactions were performed in Tris buffer (50 mM, pH 7.0) at 40°C under magnetic stirring with or without light-exposure ($I=10\%\ I_{max}$, approx. 16.8 $W.cm^{-2}$). All reactions were performed as three independent experiments (n = 3) and a representative product profile is shown. Only the final time point for the reaction with *Sm*AA10A in the dark is shown, as LPMO activity in this reaction was negligible. *Sm*AA10A activity on β-chitin was qualitatively assessed by comparing product profiles to product profile of oxidized chito-oligosaccharides with degree of polymerization ranging from 2 to 6 ($DP2^{ox}$ - $DP6^{ox}$). Product formation over time is clearly visible, despite several product peaks being partially hidden by the broad peak from kraft lignin eluting between 7 and 9 minutes as shown in the inset.

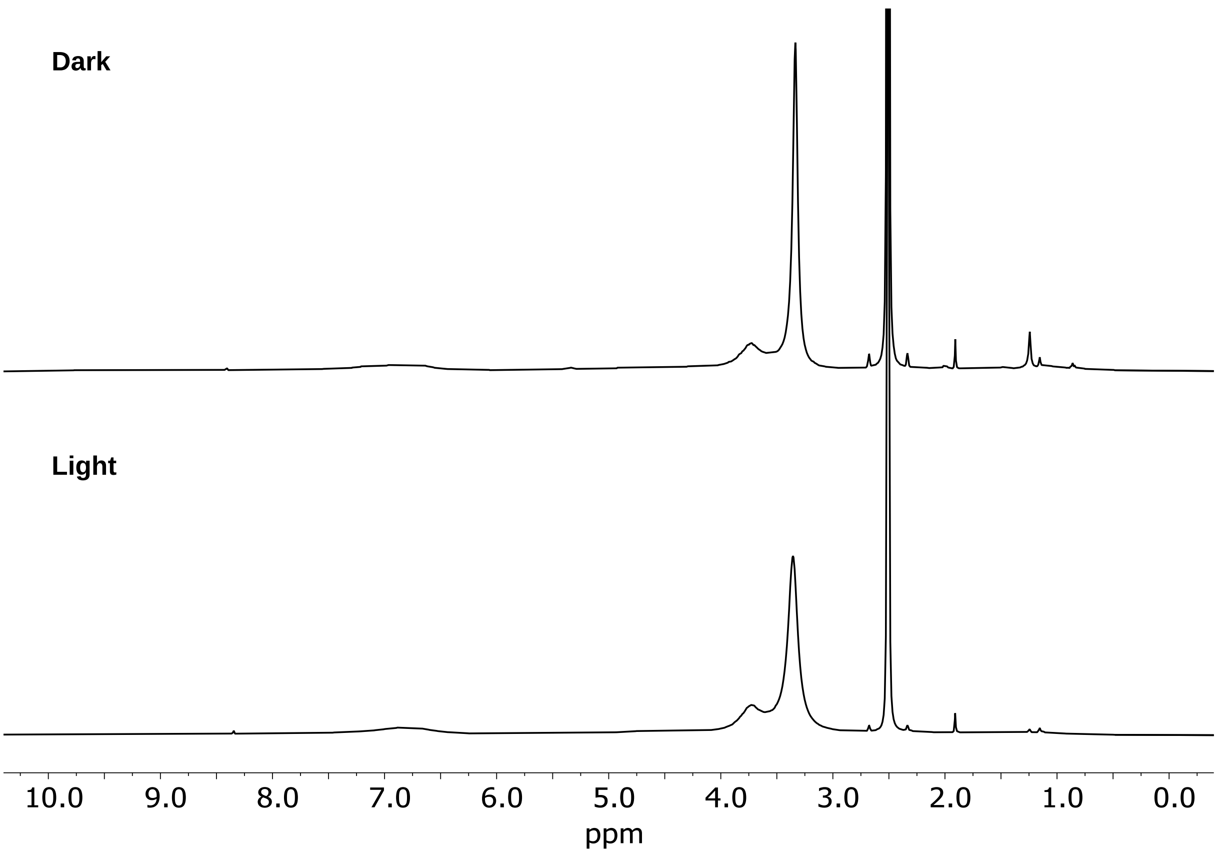

Supplementary Figure 7. Light-induced changes in kraft lignin assessed by 1D proton NMR spectroscopy. The figure shows 1D proton spectra of kraft lignin treated with light (lower spectrum) and non-treated kraft lignin (dark, top spectrum). The spectra were recorded in DMSO-d_6 (99.96 atom % D) and normalized using the peak at 3.75 ppm. Following light-exposure, the peaks at 1.23 and 3.35 ppm are reduced compared to the reference reaction in the dark. This figure is prepared from NMR data acquired with a Bruker Avance III 400 MHz spectrometer equipped with a BBFO Plus double resonance probe head at 25 °C. The 1H 1D spectra were acquired using 30-degree pulses, 8 single transients and a recycle delay of 10 s.

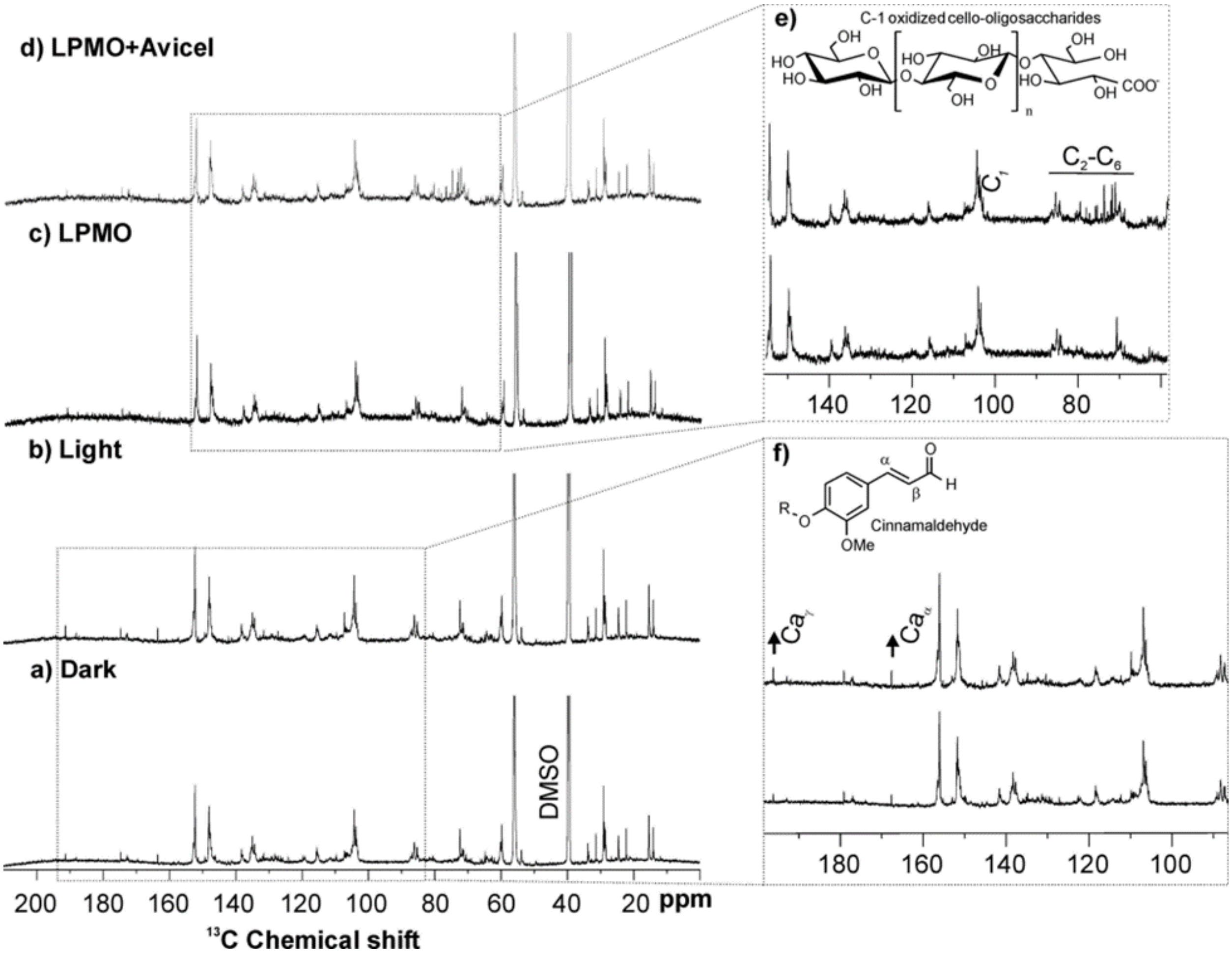

Supplementary Figure 8. Light-induced and LPMO-induced changes in organosolv birch lignin assessed by 1D carbon NMR spectroscopy. The panels show spectra obtained for organosolv lignin from birch (10 g.L^{-1}) incubated for 24 h in the dark (**a**), with light-exposure (I=10% I_{max}, corresponding to approx. 16.8 W.cm^{-2}) (**b**), in the dark with *Sc*AA10C (500 nM) (**c**), and in the dark with *Sc*AA10C (500 nM) and Avicel (10 g.L^{-1}) (**d**). Regions of the spectra displaying differences related to treatment with light (**f**) or an LMPO (**e**) are shown in the panels to the right. There were no detectable differences in the parts of the spectra that are not shown in panels **e** and **f**. All reactions were performed in sodium phosphate buffer (50 mM, pH 6.0) at 40°C with magnetic stirring. The NMR samples were prepared by dissolving either ~40 mg for light-treated lignin (**a**, **b**, **f**) or ~20 mg for LMPO-treated lignin (**c, d, e**) in 480 µL DMSO-d_6 (99.96 atom % D) and the carbon spectra were recorded at 25 °C on an 800 MHz instrument. To account for the differences in lignin concentration the intensity of all spectra was adjusted to be equal for the signal at ~28 ppm. Identification of chemical moieties, indicated in the spectra, is based on partial assignment using ^{1}H-^{13}C-HSQC and previous values reported in the literature (see Materials and Methods for more details). Signals representing the solubilised C-1 oxidized cello-oligosaccharides (C_1 [shoulder], C_2-C_6 where the number refers to the ring carbon of the monosaccharide)[2] are indicated. R indicates further coupling to the lignin polymer (**f**).

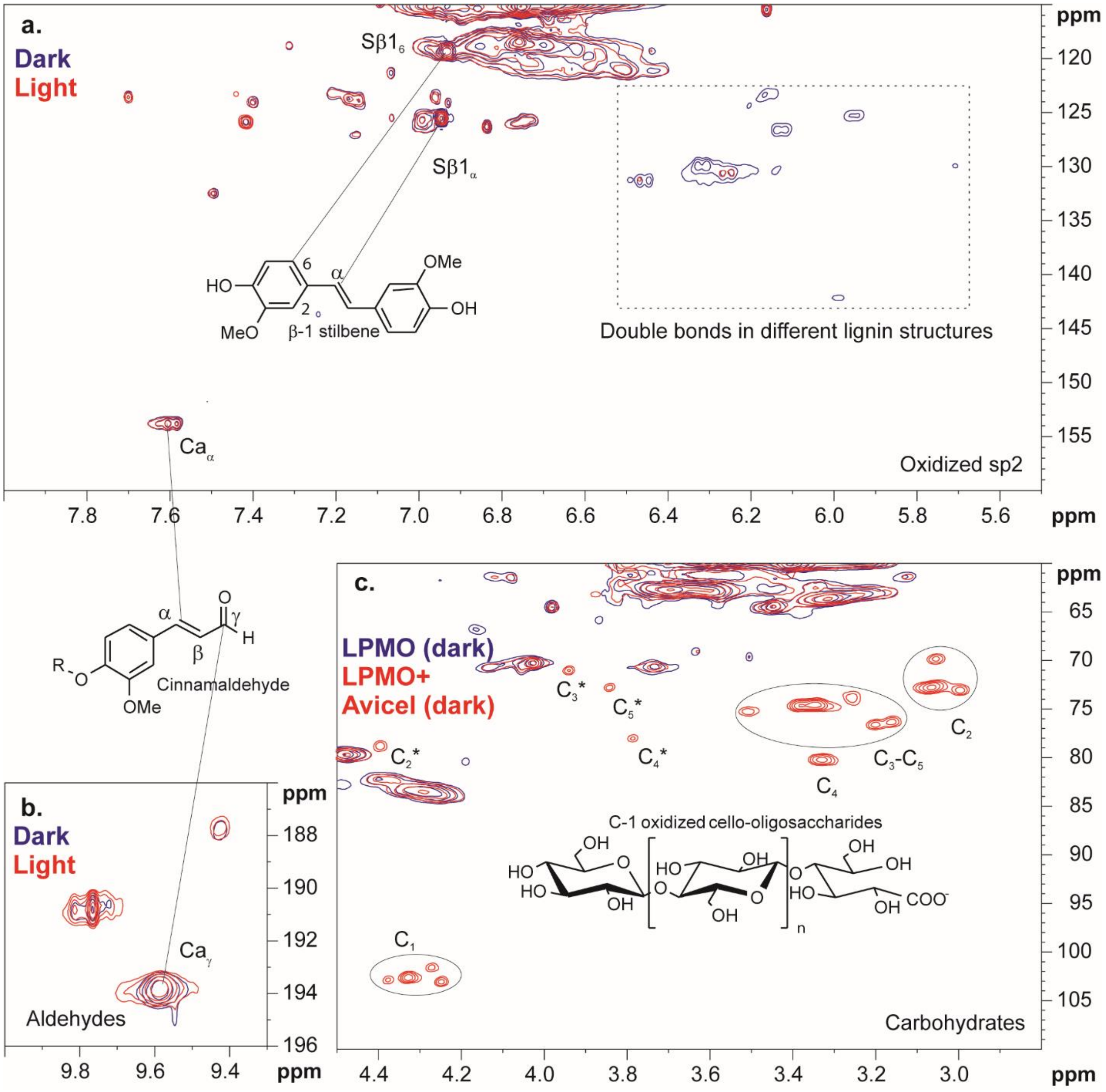

Supplementary Figure 9. **Light-induced and LPMO-induced changes in organosolv spruce lignin assessed by 2D HSQC NMR spectroscopy.** The figure shows comparisons between dark-incubated lignin (purple) and light-exposed lignin (red) for the olefinic region (**a**) and the aldehyde region (**b**). Panel (**c**) shows the region with signals from C-1 oxidized cello-oligosaccharides after incubation of lignin with LPMO (*Sc*AA10C, 500 nM) alone (cyan) or LPMO (*Sc*AA10C, 500 nM) and Avicel (10 g.L^{-1}) (red), in the dark. The NMR samples were prepared by dissolving either ~40 mg for light-treated lignin (**a**, **b**) or ~20 mg for LMPO-treated lignin (**c**) in 480 µL DMSO-d$_6$ (99.96 atom % D) and the HSQC spectra were recorded at 25 °C on an 800 MHz instrument. Identification of chemical moieties, indicated in the spectra, is based on partial assignment using ^{1}H-^{13}C-HSQC and previous values reported in the literature. Signals from β-1 stilbene (Sβ1$_\alpha$, Sβ1$_2$ and Sβ1$_6$)[3], cinnamaldehyde (Ca$_\alpha$ and Ca$_\gamma$)[3,4], and solubilised C-1 oxidized cello-oligosaccharides [C$_1$, C$_2$-C$_6$, where the number refers to the ring carbon for the monosaccharide and * indicates carbons belonging to an oxidized glucose residue[2]] are indicated. R indicates further coupling to the lignin polymer.

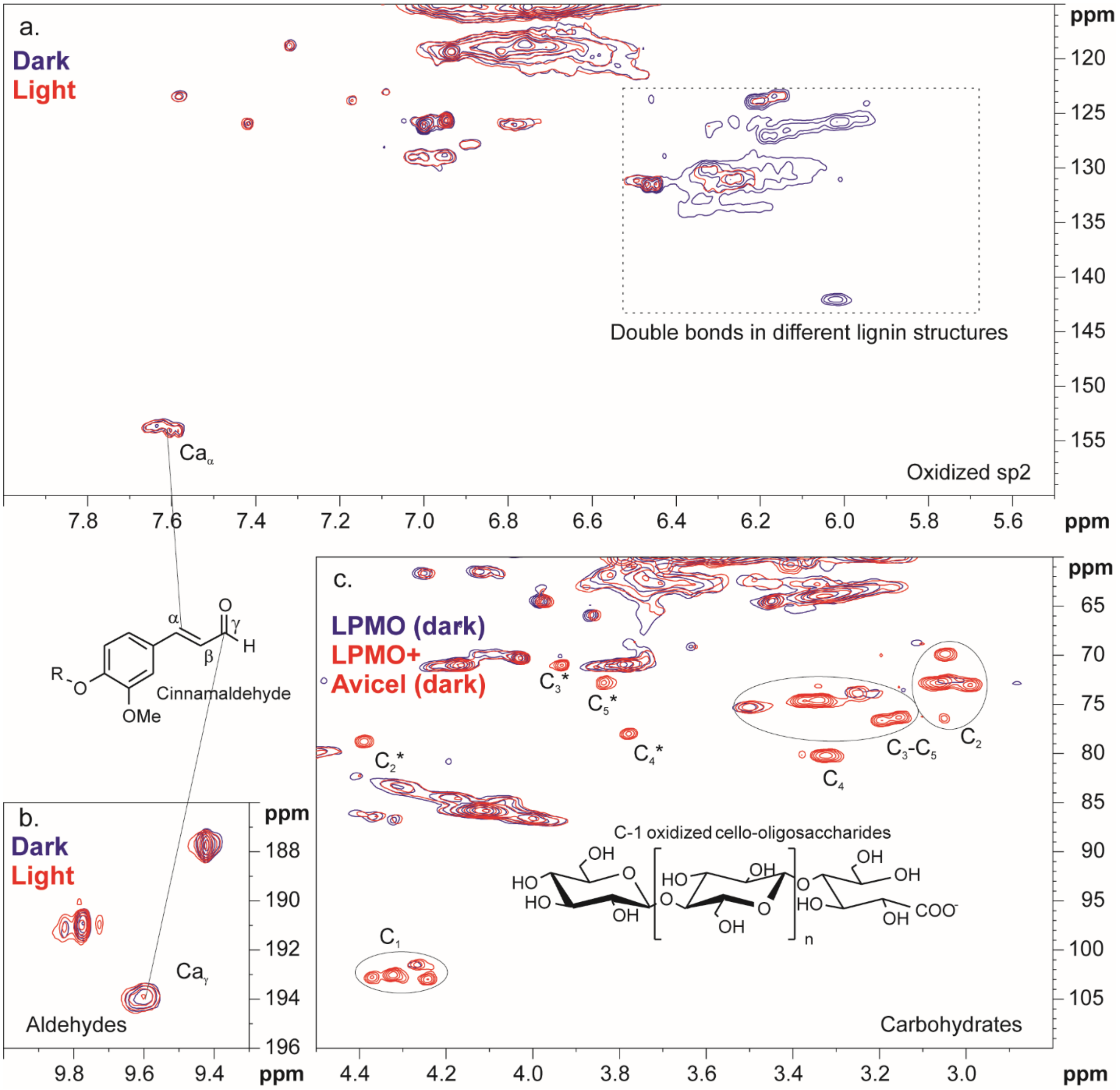

Supplementary Figure 10. **Light-induced and LPMO-induced changes in organosolv birch lignin assessed by 2D HSQC NMR spectroscopy.** The figure shows comparisons between dark-incubated lignin (purple) and light-exposed lignin (red) for the olefinic region (**a**) and the aldehyde region (**b**). Panel (**c**) shows the region with signals from C-1 oxidized cello-oligosaccharides after incubation of the lignin with LPMO (*Sc*AA10C, 500 nM) alone (purple) or LPMO (*Sc*AA10C, 500 nM) and Avicel (10 g.L^{-1}) (red), in the dark. The NMR samples were prepared by dissolving either ~40 mg for light-treated lignin (**a**, **b**) or ~20 mg for LMPO-treated lignin (**c**) in 480 µL DMSO-d$_6$ (99.96 atom % D) and the HSQC spectra were recorded at 25 °C on an 800 MHz instrument. Identification of chemical moieties, indicated in the spectra, is based on partial assignment using ^{1}H-^{13}C-HSQC and previous values reported in the literature. Signals from cinnamaldehyde (Ca$_\alpha$ and Ca$_\gamma$)[3,4] and solubilized C-1 oxidized cello-oligosaccharides [C$_1$, C$_2$-C$_6$, where the number refers to the ring carbon for the monosaccharide and * indicates the carbons belonging to an oxidized sugar residue[2]] are indicated. R indicates further coupling to the lignin polymer.

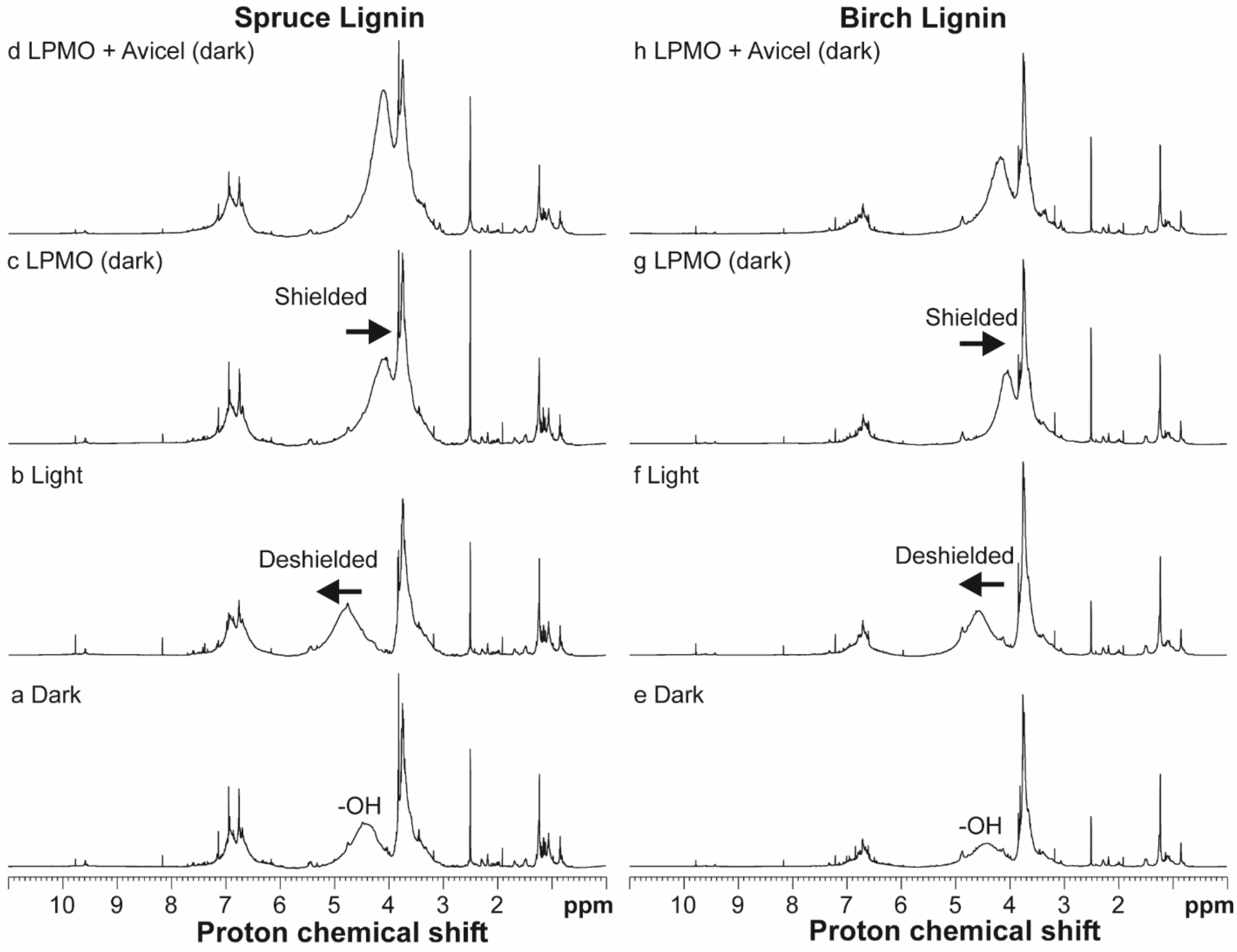

Supplementary Figure 11. Light-induced and LPMO-induced changes in organosolv birch and spruce lignin assessed by 1D proton NMR spectroscopy. The panels show spectra obtained for organosolv lignin from spruce (**a-d**) and birch (**e-h**). Lignin (10 g.L^{-1}) was incubated for 24 h in the dark (**a, e**), with light-exposure (I=10% I_{max}, corresponding to approximately 16.8 W.cm^{-2}) (**b, f**), in the dark with *Sc*AA10C (500 nM) (**c, g**), or in the dark with *Sc*AA10C (500 nM) and Avicel (10 g.L^{-1}) (**d, h**). The broad signal associated with protons of hydroxyl groups is shifted to higher frequency (deshielded) in light incubated reactions, and to a lower frequency (shielded) in reactions containing the LPMO. All reactions were performed in sodium phosphate buffer (50 mM, pH 6.0) at 40°C with magnetic stirring. The NMR samples were prepared by dissolving either ~40 mg for light-treated lignin (**a**, **b**, **e**, **f**) or ~20 mg for LMPO-treated lignin (**c**, **d**, **g**, **h**) in 480 µL DMSO-d_6 (99.96 atom % D) and the proton spectra were recorded at 25°C on an 800 MHz instrument. To account for the differences in lignin concentration the intensity of all spectra was adjusted using the lower frequency signals (δ_H 0.85, 1.24) belonging to aliphatic lignin groups that are expected to be unaffected by both light and LPMO.

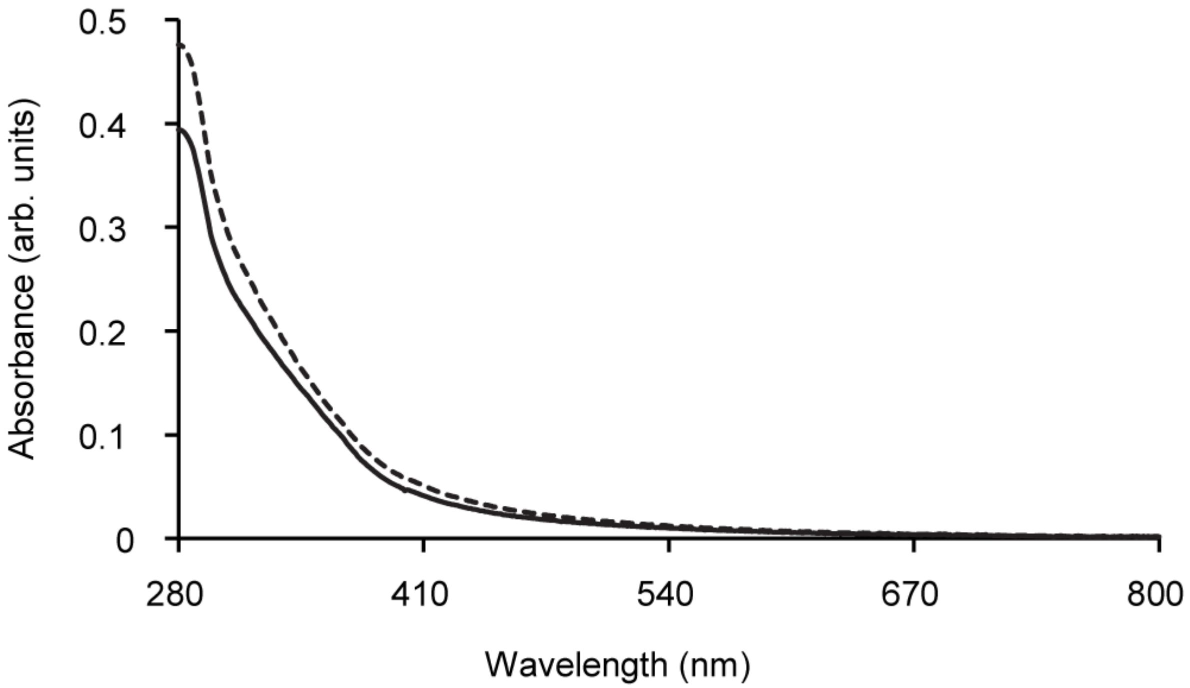

Supplementary Figure 12. UV-Vis absorption spectra of kraft lignin before and after dialysis. The figures show the absorption spectra of 0.1 g.L^{-1} native (solid line) and dialyzed (dashed line) kraft lignin. The spectra were measured in triplicates and the figure shows a representative spectrum for each lignin.

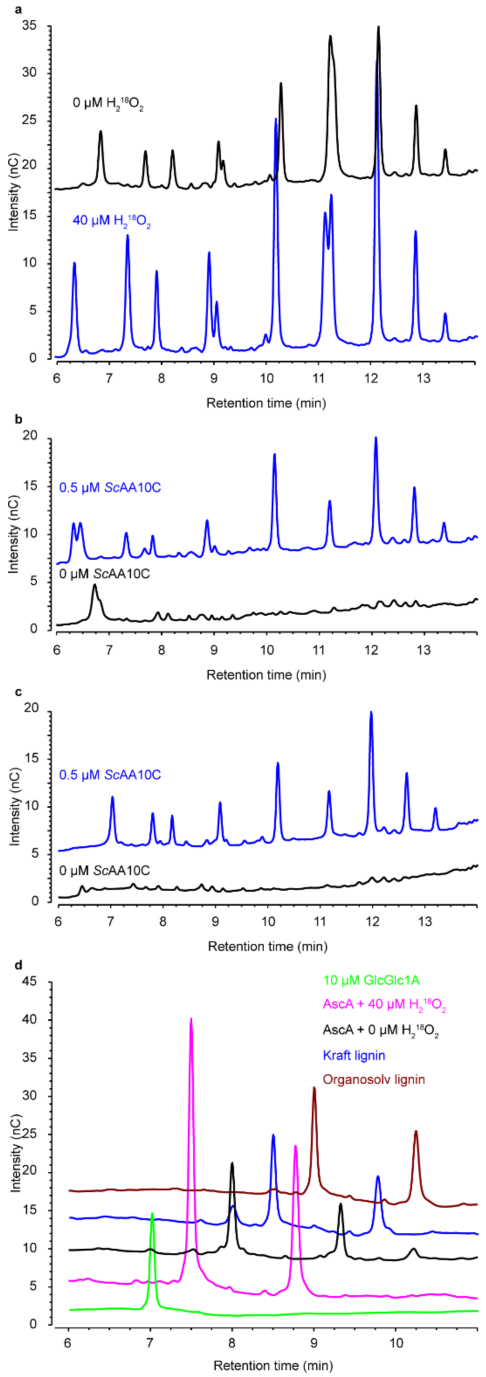

Supplementary figure 13. Chromatographic analysis of oxidized products generated in anaerobic LPMO reactions with visible light-exposed lignin. The panels show chromatographic analysis of soluble products generated in anaerobic reactions with *Sc*AA10C (500 nM) and Avicel (1 g.L^{-1}) containing 1 mM AscA with or without added ^{18}O-labelled $H_2{}^{18}O_2$ (**a**), kraft lignin (2 g.L^{-1}) (**b**), or organosolv lignin (2 g.L^{-1}) (**c**). Panel (**d**) shows the same product mixture after treatment with *Tf*Cel6A (converting oxidized products to oxidized cellobiose and cellotriose, which appear as two peaks). All reactions were conducted in ammonium bicarbonate buffer (20 mM, pH 6.9) and ^{18}O-labelled water ($H_2{}^{18}O$), and were performed in flat bottom vials with magnetic stirring, placed in an EvoluChem PhotoRedoxBox (HepatoChem) on a BioSan Mini-Shaker PSU-2T microtiter plate shaker set to 500 rpm and exposed to visible-light (EvoluChem™ LED 6200K white, with a light intensity of 29 mW.cm^{-2}) for 22 h. After 22 h, reaction mixtures were transferred to a 1.5 mL Eppendorf tube and spun down to recover the supernatants. The Eppendorf

tubes containing the supernatant were taken out of the anaerobic chamber and filtered using a 96-well filter plate (Millipore) and a vacuum manifold prior to HPAEC-PAD analysis. The samples were assessed for the presence of solubilized oxidized products before (**a**, **b**, and **c**) and, to obtain the best quantitative impression, after (**d**) treatment with *Tf*Cel6A. Product formation, at low levels, was observed in all LPMO-containing samples, but these levels were insufficient for detection using MALDI-ToF when lignin was present. As expected, higher product levels were obtained in the reaction with added H_2O_2. Importantly, panel (**d**) shows that product levels are identical for the reactions with ascorbic acid (where H_2O_2 generation through oxidation of H_2O will not occur) and the reactions with the two illuminated lignin types (where H_2O_2 generation through oxidation of H_2O might occur). This shows that in all cases the reactions are limited by the same factor, which must be the presence trace amounts of $^{16}O_2$ (i.e., the reactions were not 100 % anerobic).

We did these experiments in $H_2{}^{18}O$ and used $H_2{}^{18}O_2$ in the control reaction with hydrogen peroxide, because such an approach in principle could provide additional evidence for (the absence of) water oxidation. *Sc*AA10C-catalyzed cellulose oxidation involves hydroxylation at the C1-position of the scissile glycosidic bond to form a lactone which is in equilibrium with its hydrated form, the aldonic acid. If lignin oxidizes $H_2{}^{18}O$ to $H_2{}^{18}O_2$ and $^{18}O_2$, the aldonic acid products formed by *Sc*AA10C should display an *m/z* shift of +4 when analyzed by MALDI-TOF MS compared to products generated in a reaction with no ^{18}O present, since both oxygens in the aldonic acid would be ^{18}O. The same would be the case if oxidized products formed in reactions without added $H_2{}^{18}O_2$ would be the result of water oxidation, which would lead to in situ generation of $H_2{}^{18}O_2$. Reactions with AscA (1 mM), acting as reductant, with or without added $H_2{}^{18}O_2$ (0 or approx. 40 µM), in $H_2{}^{18}O$ and in the absence of lignin were performed as controls.

The reaction containing only AscA should not lead to any product formation in true anaerobic conditions whilst a control reaction containing AscA and $H_2{}^{18}O_2$ should provide a positive control for generation oxidized products with an *m/z* of +4. MALDI-ToF MS analyses confirmed the formation of *m/z* of +4 products in the reaction with added $H_2{}^{18}O_2$. Unfortunately, MS analysis of other reaction samples was not conclusive due to the combination of very low product levels and the presence of lignin in the samples. Aldonic acids with *m/z* +4 were not detected in these reactions. Since hardly any products with *m/z* +2 (the result of a reaction involving $^{16}O_2$) were detected neither, the formation of *m/z* +4 products cannot be excluded and the MS data, thus, do not provide additional support for the conclusions drawn from chromatographic product analysis.

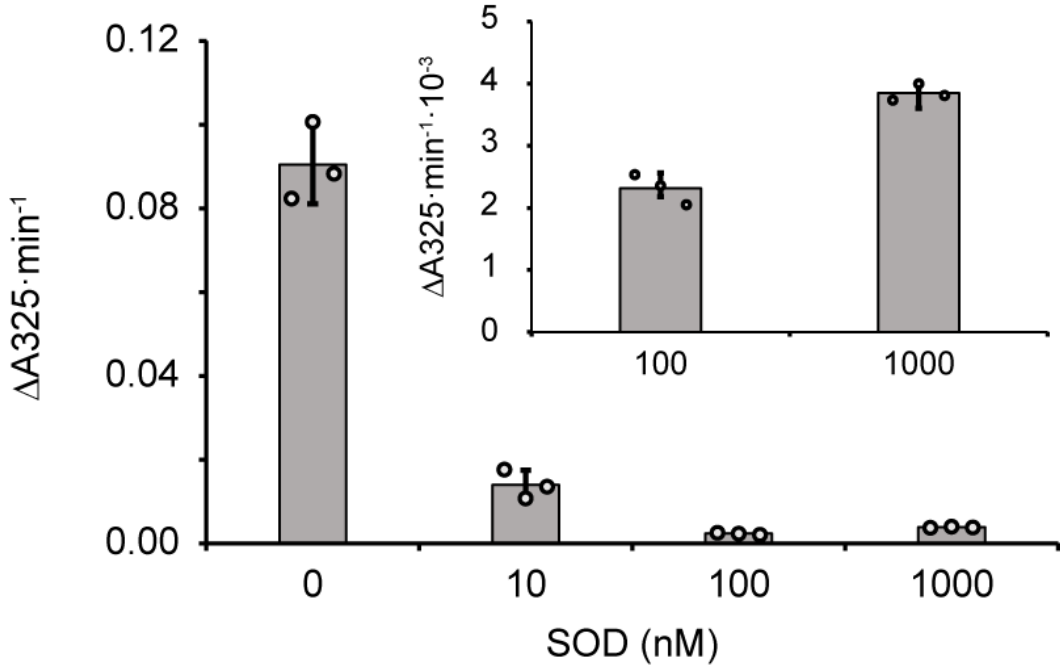

Supplementary Figure 14. Verification of Superoxide Dismutase (SOD) activity. The figure shows the change in absorbance at 325 nm during a 3-min incubation of pyrogallol leading to its autooxidation to purpurogallin, and how increasing amounts of SOD inhibit this reaction. At alkaline pH and aerobic conditions, autooxidation of pyrogallol leads to formation of superoxide radicals that drive formation of purpurogallin, and the latter can be spectrophotometrically measured at 325 nm. Adding SOD removes superoxide and inhibits formation of purpurogallin. The rate was derived using all data points from the 3-min reaction using linear regression. R^2 was > 0.98 for reactions with 0 and 10 nM SOD, while for reactions with 100 and 1000 nM SOD R^2 was > 0.7. The data is presented as mean values and error bars show ± s.d. (n = 3, independent experiments).

Supplementary references

1. Westereng, B. *et al.* Simultaneous analysis of C1 and C4 oxidized oligosaccharides, the products of lytic polysaccharide monooxygenases (LPMOs) acting on cellulose. *J. Chromatogr. A* 46–54 (2016). doi:10.1016/j.chroma.2016.03.064

2. Westereng, B. *et al.* Efficient separation of oxidized cello-oligosaccharides generated by cellulose degrading lytic polysaccharide monooxygenases. *J. Chromatogr. A* **1271**, 144–152 (2013).

3. Lancefield, C. S., Wienk, H. L. J., Boelens, R., Weckhuysen, B. M. & Bruijnincx, P. C. A. Identification of a diagnostic structural motif reveals a new reaction intermediate and condensation pathway in kraft lignin formation. *Chem. Sci.* **9**, 6348–6360 (2018).

4. Ralph, S. A., Ralph, J. & Landucci, L. L. NMR Database of Lignin and Cell Wall Model Compounds. (2009).
